# Identification of a TP53 Mutation in a Patient With Li-Fraumeni Syndrome and Not Meeting the Revised Chompret Criteria: A Case Report

**DOI:** 10.7759/cureus.40025

**Published:** 2023-06-06

**Authors:** Monika Iwasaki, Clara So, Torahiko Jinta

**Affiliations:** 1 Department of Pulmonary Medicine, Thoracic Center, St. Luke's International Hospital, Tokyo, JPN

**Keywords:** brca2, braca1, her2-positive, heritable tp53-related cancer syndrome, multiple cancer, lung cancer, breast cancer, tp53, chompret criteria, li-fraumeni syndrome

## Abstract

Li-Fraumeni syndrome (LFS) is a rare familial disorder caused by germline TP53 mutations. Despite the establishment of the revised Chompret criteria to guide genetic testing for TP53, identifying LFS in patients who do not satisfy these criteria remains a challenge. Herein, we present the case of a 50-year-old woman with a history of breast, lung, colorectal, and tongue cancers who did not satisfy the revised Chompret criteria. However, genetic testing ultimately revealed a TP53 mutation, leading to the diagnosis of LFS. Although her family history did not satisfy the classic LFS criteria, she had a TP53 core tumor before the age of 46 years. This case highlights the importance of considering LFS in patients with a history of multiple cancers and suggests that genetic testing should be considered even in patients who do not satisfy the revised Chompret criteria.

## Introduction

Li-Fraumeni syndrome (LFS) is a rare familial disorder caused by a germline mutation in the TP53 gene. Definitive diagnosis requires the detection of a germline TP53 mutation [1]. However, some patients who present with familial cancer predisposition without TP53 mutation satisfy the classic LFS diagnostic criteria [2]. Approximately 70%-75% of patients with LFS will have a mutation in the TP53 gene. Nevertheless, some patients who present with familial cancer predisposition without TP53 mutation satisfy the classic LFS diagnostic criteria [2-4]. According to the Classic LFS diagnostic criteria, patients must satisfy all of the following criteria. First, the proband had an onset of sarcoma before the age of 45 years. Second, the first-degree relatives developed cancer before the age of 45 years. Third, the first- and second-degree relatives were diagnosed with cancer before the age of 45 years or developed sarcoma regardless of age [2]. TP53 mutations have also been observed in individuals without a family history of cancer. Therefore, the use of a more inclusive term, 'heritable TP53-related cancer syndrome', has been proposed recently [1,5]. The revised Chompret criteria are widely used as a guide to determine when to test for TP53. As per the criteria, it is recommended to conduct testing in the following scenarios: a proband with a TP53 core tumor (breast cancer, soft-tissue sarcoma, osteosarcoma, central nervous system tumor, adrenocortical carcinoma) before the age of 46 years and at least one first- or second-degree relative with a core tumor before the age of 56 years (other than breast cancer if the proband has breast cancer); a proband with multiple primary tumors including two TP53 core tumors, the first of which developed before the age of 46 years, irrespective of family history; and patients with adrenocortical carcinoma, choroid plexus carcinoma, or rhabdomyosarcoma of embryonal anaplastic subtype, irrespective of family history, or breast cancer before the age of 31 years, irrespective of family history [6]. In addition, the National Comprehensive Cancer Network (NCCGN) suggests genetic testing for people with negative BRCA1/2 mutation as they have higher rates of TP53 mutations [5]. It is also reported that human epidermal growth factor receptor-2 (HER2)-positive breast cancer is associated with TP53 mutations [5]. Although genetic testing has been widely used in recent years, it is still difficult for patients and doctors to decide whether genetic testing would be beneficial [4]. We present a rare case of LFS that did not satisfy the revised Chompret criteria but revealed TP53 mutation on genetic testing.

## Case presentation

**Figure 1:**
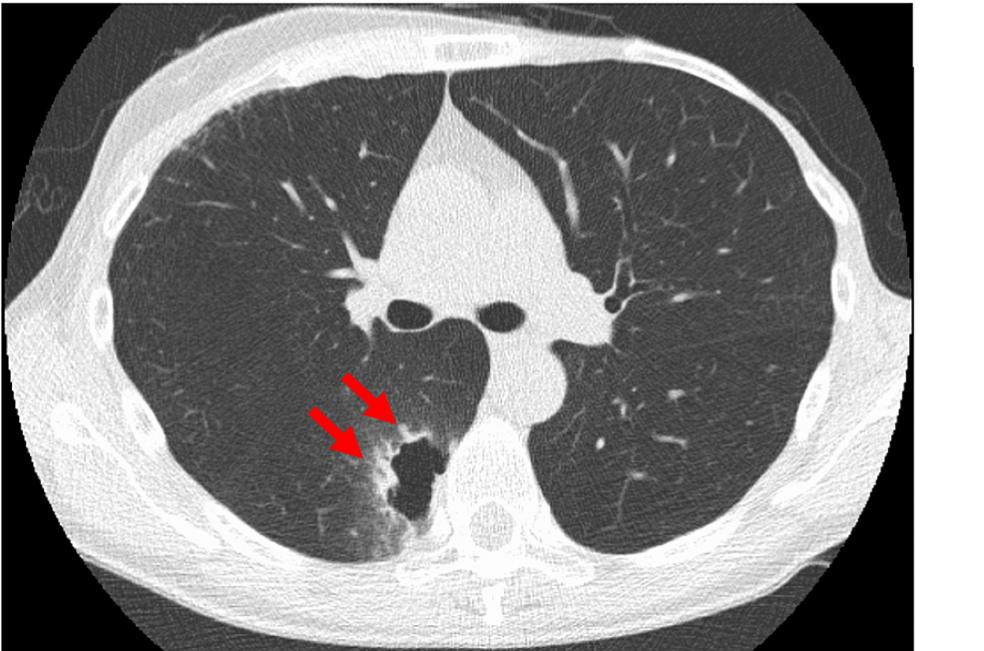
CT image at the time of diagnosis of right lung cancer This is the CT image of right lung cancer with a cavitation which is pointed by the arrows.

A 50-year-old woman with a history of bilateral breast cancer was referred to our department for the treatment of right lower lobe lung cancer. The right lung cancer, with a cavity, was detected during follow-up computed tomography (CT) after radiation therapy for right breast cancer (Figure 1). She had been diagnosed with estrogen, progesterone, and HER2 triple-positive left breast cancer at the age of 31 years. It was treated by partial left mastectomy with lymph node dissection and adjuvant therapy.

**Figure 2:**
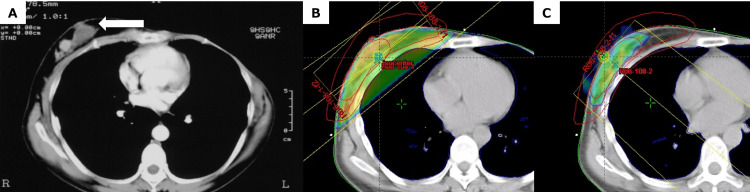
CT image at the time of diagnosis of right breast cancer, and radiation therapy field after the surgery for right breast cancer In Figure A, the arrow (←) shows the right breast cancer, after mastectomy was performed on left breast cancer. Figure B and C shows the radiation field after surgery for right breast cancer. In Figure B, the colored area shows the tangential irradiation field. In Figure C, the colored area shows the boost irradiation field.

**Figure 3:**
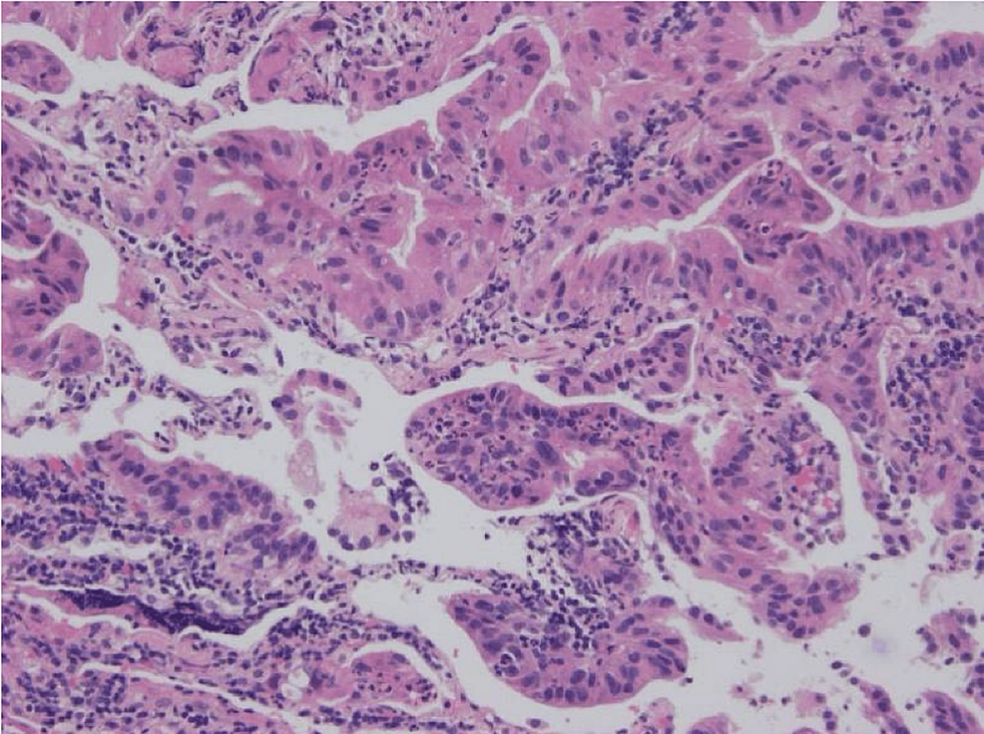
Pathological image of right lung cancer This is a pathological image of the right lung cancer which shows invasive mucinous adenocarcinoma. Cells with nuclear enlargement and nuclear atypia are proliferating irregularly, forming glandular structures in a cystic pattern.

Subsequently, she was diagnosed with estrogen-negative, progesterone-negative, and HER2-positive right breast cancer at the age of 44 years. She underwent a right partial mastectomy and received external-beam radiation therapy to the right breast (Figures 2A-2C). Genetic testing for BRCA1 and BRCA2 yielded negative results. After referral to our department, she underwent a right lower lobectomy and was diagnosed with right lung cancer with pT4N0M0 stage IIIA invasive mucinous adenocarcinoma (Figure 3). The tumor recurred at the age of 52 years and was tested for genetic mutations. Real-time polymerase chain reaction (AmoyDx) revealed that the lung biopsy sample was negative for genetic mutations and PD-L1. At the ages of 52 and 56, she was diagnosed with colorectal and tongue cancer, respectively. The colorectal cancer, classified as type 0-Is, was situated in the ascending colon. Pathological examination revealed a well-differentiated tubular adenocarcinoma with a stage of pTisNXM0. She underwent endoscopic mucosal resection, resulting in complete removal of the cancer. The pathology results ruled out the possibility of the lung cancer being a metastasis of the colon cancer. Her tongue cancer was suspected based on cytology, indicating class IIIA, and it was closely monitored. Given her history of multiple cancers, we suspected a familial predisposition to cancer. Her family history was as follows: her father had a history of colorectal cancer in his 60s, her aunt had a history of breast cancer in her 30s, and her cousin had a history of a brain tumor at the age of 13 years. Although she did not satisfy the classic LFS diagnostic criteria or the revised Chompret criteria, we conducted genetic testing at the age of 53 years. TP53 mutation with a deletion on exon 6 C.586C. T(p.Arg196*) was revealed, resulting in the diagnosis of LFS. None of the family members with a history of cancer were alive at the time of TP53 testing; therefore, they did not undergo genetic testing. She did not have any siblings or children. Recurrence after right lower lobectomy for lung cancer was treated with chemotherapy; however, she died at the age of 61 years due to exacerbation of the lung cancer.

## Discussion

LFS is typically regarded as a familial disorder, and its identification is challenging in the absence of any familial history. Although our patient had a TP53 core tumor before the age of 46 years, she did not satisfy the revised Chompret criteria due to the absence of first- or second-degree relatives with a core tumor before the age of 56 years. Although her aunt had a history of breast cancer in her 30s, this constituted an exclusion criterion since the proband had breast cancer. The patient did not satisfy the classic LFS criteria as she did not develop sarcoma.

In the present case, genetic testing for TP53 was conducted more than two decades after the patient’s initial diagnosis of left breast cancer. In retrospect, the only possible indications for earlier testing of TP53 were HER2 positivity and negative BRCA1/2 mutation, which have been suggested to be associated with TP53 mutations. However, approximately 90% of patients with breast cancer have negative BRCA1/2 results [7]. Thus, negative BRCA1/2 results alone may not necessarily lead to the decision to conduct TP53 genetic testing. In contrast, HER2 positivity is present in 19% of breast cancer patients below the age of 49 years in the U.S., which may create a dilemma regarding whether to conduct genetic testing for all HER2-positive patients [8]. Although we were able to conduct genetic testing for TP53 due to the history of multiple cancers, our case highlights the importance of considering genetic testing for TP53 even if the revised Chompret and classic LFS diagnostic criteria are not satisfied. The limitation of this case lies in its inability to encompass the entirety of LFS cases that do not meet the criteria. However, even in light of this limitation, we strongly emphasize the importance of conducting TP53 testing in patients who are diagnosed with HER2-positive breast cancer and negative results for BRCA1/2 mutations. Earlier detection of TP53 mutation is crucial as it will aid in avoiding radiation therapy which could result in secondary neoplasms, and in initiating constant surveillance for other cancers [9].

## Conclusions

The revised Chompret criteria are useful for determining when to perform genetic testing for TP53 in patients with cancer; however, clinicians should be aware of cases that do not satisfy these criteria. We report a case of LFS with a TP53 mutation without significant family history that did not satisfy the revised Chompret and classic LFS diagnostic criteria. Our patient had a history of breast cancer with HER2-positivity and BRCA1/2 negative results which have been linked to TP53 mutation. Although it is not considered mandatory to test for TP53 in HER2-positive and BRCA1/2 negative cases, we strongly recommend considering TP53 genetic testing and surveillance for any other cancers.
